# Halophilic Archaea Cultivated from Surface Sterilized Middle-Late Eocene Rock Salt Are Polyploid

**DOI:** 10.1371/journal.pone.0110533

**Published:** 2014-10-22

**Authors:** Salla T. Jaakkola, Karolin Zerulla, Qinggong Guo, Ying Liu, Hongling Ma, Chunhe Yang, Dennis H. Bamford, Xiangdong Chen, Jörg Soppa, Hanna M. Oksanen

**Affiliations:** 1 Institute of Biotechnology and Department of Biosciences, University of Helsinki, Helsinki, Finland; 2 Institute for Molecular Biology, Goethe-University Frankfurt am Main, Frankfurt am Main, Germany; 3 State Key Laboratory of Virology, College of Life Sciences, Wuhan University, Wuhan, Hubei, People's Republic of China; 4 Institute of Rock and Soil Mechanics, The Chinese Academy of Sciences, Wuhan, Hubei, People's Republic of China; Hellas, Greece

## Abstract

Live bacteria and archaea have been isolated from several rock salt deposits of up to hundreds of millions of years of age from all around the world. A key factor affecting their longevity is the ability to keep their genomic DNA intact, for which efficient repair mechanisms are needed. Polyploid microbes are known to have an increased resistance towards mutations and DNA damage, and it has been suggested that microbes from deeply buried rock salt would carry several copies of their genomes. Here, cultivable halophilic microbes were isolated from a surface sterilized middle-late Eocene (38–41 million years ago) rock salt sample, drilled from the depth of 800 m at Yunying salt mine, China. Eight unique isolates were obtained, which represented two haloarchaeal genera, *Halobacterium* and *Halolamina*. We used real-time PCR to show that our isolates are polyploid, with genome copy numbers of 11–14 genomes per cell in exponential growth phase. The ploidy level was slightly downregulated in stationary growth phase, but the cells still had an average genome copy number of 6–8. The polyploidy of halophilic archaea living in ancient rock salt might be a factor explaining how these organisms are able to overcome the challenge of prolonged survival during their entombment.

## Introduction

Cultivable bacteria and archaea have been isolated from rock salt up to hundreds of millions of years of age (MYA), representing species of *Haloarcula*, *Halobacterium*, *Halococcus*, *Haloterrigena*, *Natronobacterium*, *Natronomonas*, and *Virgibacillus* [1]–[9]. These findings have inspired discussion about the theoretical maximum age an organism can reach, and their possible tactics for survival [10]–[12]. The first microbes isolated from halite burial sites (see review by Kennedy *et al.* [13]) were dismissed as probable laboratory contaminants, based on their similarity to known organisms [14]. More recently, rigorous chemical surface sterilization [15], [16], brine extraction from liquid inclusions in halite with microscopic drills [1], [7], as well as microscopic identification of cells inside the crystals prior to extraction [7], [17] have been used to detect cells inside the halite, and to minimize the possibility of contamination. An additional argument that the isolated haloarchaea are not systematic contaminations is the fact that the isolation of living cells is typically only successful for a minor fraction of the samples that were taken at different sites of halite deposits. Furthermore, dating of liquid inclusion brine has also provided substantial evidence of the age of the isolates [18], [19]. Many independent studies have repeated the isolation of viable microbes from ancient halite samples from globally separated sites in Austria, Chile, UK, and US [5], [6], [8], [9]. Postsedimentary origins have also been suggested for this type of isolates based on the assumed maximum age for intact DNA [20]. However, no geological evidence for microbial contamination of deeply buried strata has been presented.

The idea of microbes overcoming extremely long periods of time has not been accepted without disbelief. When discussing the possibility of viable microbes surviving inside bedded halite, the main concern has been degradation of DNA due to radiation and hydrolysis [20], [21]. It has been suggested that the microbes regress into a state of minimal metabolism, where only vital functions are maintained [22], which would allow cells to repair DNA damage. Evidence for active metabolism and DNA repair has been obtained from bacteria inside permafrost [23]. In some studies, small, round, spore-like cells have been observed inside salt crystals and ice core samples [17], [24]–[26]. Some halophilic archaea, such as *Halobacterium salinarum*, *Halobacterium noricense*, and *Haloferax mediterranei* adopt a spherical form inside halite crystals [27]. These spherical cells, which have a lowered ATP content and frequently form clusters, are apparently resting forms of these otherwise rod-shaped or polymorphic archaea [27].

It has been suggested, that polyploidy could be one factor positively affecting the ability of a microbe to remain viable while being encased in halite [28]. Both bacteria and archaea can be polyploid [29], [30]. Some species, such as *Caulobacter crescentus* and *Wolinella succinogenes*, are monoploid, whereas others may carry several identical copies of their genomes [30]. The best characterized gram-negative and gram-positive bacteria, *Escherichia coli* and *Bacillus subtilis*, are only monoploid when grown with very low growth rates, but are mero-oligoploid when grown under optimal conditions [30, Böttinger *et al.*, in revision]. Genome copy numbers in polyploid species vary over a large scale. The copy number in symbiotic bacteria Candidatus *Sulcia muelleri* and *Epulopiscium fishelsoni* are around 200–900 and 50,000–120,000, respectively [31], [32]. A ploidy level of fifty-five has been detected in *Methanococcus maripaludis*, which is the current record among archaea [33]. Polyploidy seems to be common in euryarchaeal species, in contrast to the members of Crenarchaea, which are monoploid with no known exception [34].

Having more than one chromosome is advantageous to a microbe in many ways [28]. Multiple replicons might enable global regulation of cell functions, and gene dosage can be increased by upregulating the level of ploidy. In actively dividing cells, the genome copy number is typically higher than that in stationary phase [34]. As a relatively stable molecule, DNA can also serve as a storage polymer of phosphate [35], which is often a limiting factor in microbe growth [36]. Importantly, the ability of a polyploid organism to counteract mutations and DNA damage is enhanced, as the undamaged copies serve as templates for repair [28]. The mutation rates of polyploid prokaryotic species seem to be lower than those of monoploid ones, as has been shown for *Haloferax volcanii* [37]. Some methanogenic archaea and halophilic *Hfx. volcanii* use gene conversion by homologous recombination to keep all of their chromosome copies identical, effectively neutralizing mutations [33], [38]. Radiation results in double strand breakages in DNA, which are difficult to repair. The extremely radiation-resistant bacterium *Deinococcus radiodurans* and archaeon *Halobacterium salinarum* are both polyploid [29], [39]. They are able to restore their genomes after radiation or desiccation induced double strand breaks within 24–29 hours [29], [40], which is believed to be related to their polyploid nature [28]. Oligoploid cells of *Escherichia coli* have also an enhanced resistance towards double strand breakage compared to monoploid ones [41].

In this study, we cultivated viable cells from a surface sterilized salt crystal originating from a middle-late Eocene halite formation in Yunying salt mine, China. Eight phenotypically different isolates of halophilic archaea were obtained, representing *Halobacterium* and *Halolamina*, based on their partial 16S rRNA gene sequences. The fastest growing representatives of both genera were used for ploidy level analysis by real-time PCR [29]. Our results indicate that polyploidy is common in buried halophilic archaea, and possibly helps them preserve their genomes in a functional state for a prolonged time.

## Geological Settings

**Figure 1:**
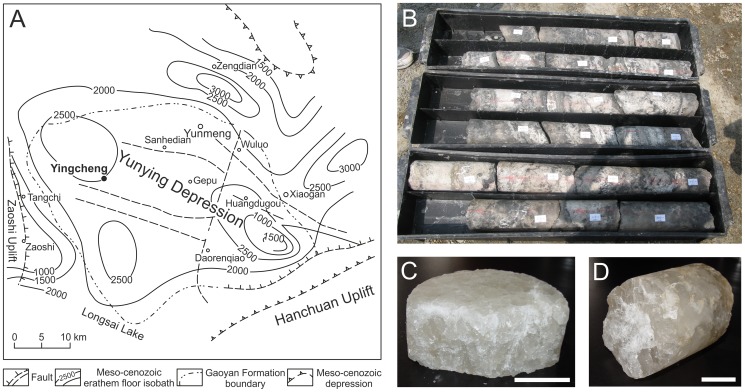
Sampling location and the drill core sample. (A) The map of Yunying Depression, with the sampling site, Yingcheng (30° 55′ N, 113°34′ E), bolded and marked with a black dot. (B) Subsections of a drill core from the site. (C) The 600 m (D) and the 800 m samples, of which subsections were used for microbe isolation in this work. The bars in C and D represent 5 cm.

The Yunying Depression (Fig. 1A) in Hubei Province, China, is a well-documented geological formation with halite deposits located in the northeastern part of the Jianghan Basin. It has formed during the Cretaceous and Early Paleogene periods and has an area of approximately 4500 km^2^ [42]. The depression is a faulted inland saline lake basin, unaffected by marine waters [43]. It consists of seven different formations, of which four (Wenfengta, Gaoyan, Baishakou, and Yuntaishan) have formed during the Paleogene, 23–66 MYA [43]. Rock salt deposits in the Yunying Depression are of Paleogene origin and cover an area of around 260 km^2^ [44].

The Yunying salt mine resides in the middle of the Yunying Depression, in the city of Yingcheng. Most of the salt is mined from the Gaoyan Formation, which has a thickness between 394 to 1598 meters and is divided into five different layers: the lower anhydrite, the lower glauberite, halite, the upper glauberite, and the upper anhydrite layer [44]. The Gaoyan formation has formed during the middle-late Eocene, around 38–41 MYA [45]. Sedimentation in this area has been rhythmic due to alternating dry and wet seasons, and salt layers are separated by layers of mudstone [44]. Abundant chevron crystals are seen in the halite layers [45]. The temperature of the liquid inclusion brine inside Gaoyan halite at the time of salt crystallization has been measured, and it is somewhat higher than the current temperatures in the area during the dry period, but correlate well with the estimated temperatures of the dry seasons during Eocene, suggesting the inclusions have formed near surface and are primary [45]. The mineral layers in Yunying salt mine have a high mechanical integrity and low permeability [46], inhibiting outside contamination.

## Materials and Methods

### Sampling site

Rock salt drill core samples from the depths of ∼600 and 800 m (79 g and 106 g, respectively) were obtained from Yunying salt mine (30° 55′ N, 113°34′ E) (Fig. 1A) in Hubei Province, China, by China National Petroleum Corporation (CNPC) who is in charge of the drilling activity in this area. There are no endangered or protected species in the salt mine. All core samples in the study were under the permits of China National Petroleum Corporation (Y115151G01). The mine resides in Yunying Depression, which is the northeastern part of Jianghan Basin (see above Geological settings) [43].

### Surface sterilization and microscopy of the crystals

For crystal sterilization, a cell cultivation room with an entry chamber was used, where no halophiles had been handled before. We used a laminar flow hood cleaned with antimicrobial solution [0.06% (w/v) cocospropylendiaminguanidindiacetat, 0.08% (w/v) didecyloxyethylmethylammoniumpropionat, PAN Biotech GmbH, PAN Biotech GmbH] and 70% ethanol in succession before and after crystal handling. All solutions and equipment were autoclaved prior to use. Samples of the solutions used in the sterilization process, excluding NaOH and HCl, were plated on MGM plates and incubated for up to three months to confirm the effectiveness of the autoclaving (for plating conditions, see Microbe isolation and cultivation). A new set of clean protective clothing was used for handling of individual crystals.

Surface sterilization of the crystals was carried out along the method of Rosenzweig *et al.* [16] with some modifications. The crystals were rinsed twice in sterile water to dissolve the outer layer and smoothen up the surface for efficient sterilization. This was followed by incubation in 10 M NaOH (5 min), saturated NaCl (1 min), 10 M HCl (5 min), saturated NaCl in 100 mM NaCO_3_ (2 min), and sterile water (2×1 min). Dry surface sterilized crystals were cut in half, and final samples were chiseled off from the fresh crystal surface and placed into sterile pre-weighed tubes. Several subsamples were taken from the crystals.

Fragments cut from the inside of surface sterilized salt crystals were examined by light microscopy (Nikon SMZ745T microscope, Jenoptik ProgRes SpeedXT Core 3 camera) to visualize crystal structure and liquid inclusions.

### Microbe isolation and cultivation

Halite samples were dissolved {1 g sample/1 ml 20% artificial salt water (SW), containing 2.7 M NaCl, 98 mM MgCl_2_, 95 mM MgSO_4_, 63 mM KCl, 3.3 mM CaCl_2_, and 53 mM Tris-HCl pH 7.2 [47], [48]} and spread on modified growth medium (MGM) [47], [48] plates with 20% SW using sterile disposable equipment, followed by incubation at 37°C for up to three months. We used new plastic boxes with lids to protect the plates from drying and external contaminants. Cups of sterile water were kept inside the boxes to maintain humidity. The plating was repeated using another subsample to get consistent results. Phenotypically different colonies were chosen from the plates, and pure cultures of isolates were obtained by three consecutive platings of a single colony. The unique isolates were named in order of isolation from YI80-1 to YI80-8.

The cells were grown aerobically at 37°C in MGM broth or on MGM plates [47], [48] with 23% or 20% SW, respectively. MGM contained 5 g peptone (Oxoid) and 1 g yeast extract (BD) per liter, and 14 g/l of agar (BD) was added to obtain MGM plates.

### Microscopy, and protein pattern and phylogenetic analyses of the isolates

Sodium dodecyl sulfate polyacrylamide gel electrophoresis (SDS-PAGE) [14% (w/v) acrylamide] and Coomassie blue staining were used to analyze the major proteins of entire cells [49]. Late stationary cell cultures in MGM broth were examined by light microscopy (Olympus BX50F-3).

Partial 16S rRNA gene sequences were amplified by PCR as previously described [50]. Primers D30 and D56 [51] were used for all strains except for YI80-6, YI80-7, and YI80-8, for which D30 and PV6-1 [52] were used, as their 16S rRNA gene sequences could not be amplified with D56. Primers D30, D56, PV6-1, pD, and pDr [53] were used for sequencing of the PCR products at the Institute of Biotechnology, DNA Sequencing and Genomics Laboratory (University of Helsinki). The 16S rRNA gene sequences of our isolates were compared to the EzTaxon-e database using BLASTN at http://www.ezbiocloud.net/eztaxon [54].

Alignment of sequences was performed with ClustalW [55]. Maximum likelihood and maximum parsimony phylogenetic trees were generated by Molecular Evolutionary Genetics Analysis (MEGA) software version 5.05 [56], with 1000 bootstrap samplings. Trees were first constructed with all type species of Halobacteriaceae and members of *Halobacterium* and *Halolamina*. Subsections of the original trees were used to create the final trees. *Methanospirillum hungatei* was used as an outgroup.

### Ploidy level analysis

The real-time PCR method for ploidy determination requires the presence of sequence information [29]. No sequence information of the new isolates was available apart from the 16S rRNA gene sequence, which could not be used because the copy numbers of the ribosomal RNA operons of the isolates were unknown and many species contain more than one copy. Therefore, we generated sequence information of the *radA* gene, which encodes the DNA repair and recombination protein RadA. The *radA* gene was chosen, because it is present in all species in all three domains of life, (*radA* in archaea, *recA* in bacteria and *rad51* in eukaryotes). The RadA protein is involved in an early step of homologous recombination, which is essential for replication as well as for DNA repair. In addition, *radA* (or *recA*) is always a single-copy gene and thus ideally suited for the quantification of ploidy levels. Furthermore, the degree of conservation is very high, which enables the amplification of a large portion of unknown *radA* gene with degenerate primers of two sites of especially high conservation. The *radA* sequences of eight archaeal species were retrieved from the HaloLex database (www.halolex.mpg.de) and a multiple sequence alignment was generated using ClustalW. Four highly conserved regions were used to design the degenerate oligonucleotides (Table 1). The oligonucleotides were used for amplification and sequencing of a *radA* fragment of about 1 kbp using standard PCR with cell extracts of the three isolates YI80-1, YI80-4 and YI80-5 as templates. For the preparation of the cell extracts, aliquots of 3×10^8^ cells were harvested by centrifugation (5 min, 13000 rpm) and resuspended in 100 µl of 20% SW. The cells were lysed by addition of 900 µl sterile water, and 1 µl of the corresponding cell extract was used for PCR reaction. The PCR fragments were purified and sequenced from both sides using the degenerate oligonucleotides as primers.

**Table 1:** Oligonucleotides used in this study.

Isolate	Oligonucleotide	5′- 3′	Application
YI80-1, YI80-4, YI80-5	*radA*-for	ctsccsggygtbgghccvgcdachgcvgav	amplification and sequencing of the *radA* gene
	*radA*-rev	tgsaggtgyttgttgagyttctgctg	
YI80-1	standard for	ggagtccgggttggaggcgacctg	standard fragment
	standard rev	gacggcggagaaactcgaagacaacgg	
	analysis for	gcttgcgatttcctgggccttctcgg	analysis fragment
	analysis rev	gctctcggtcaacgtccagttaccggcc	
YI80-4	standard for	cggggttggaggcgacctggttgg	standard fragment
	standard rev	cggccgaacgtgatcaaacgcgg	
	analysis for	ccttcgcgagttcgagggccttctcg	analysis fragment
	analysis rev	ccaggtcacccaccagatggcggtc	
YI80-5	standard for	gcttgttgagcttctgctgtcgctcggc	standard fragment
	standard rev	ccgccgacaagctcgtcgacgc	
	analysis for	cgtgttccttcgcgagttcgagggc	analysis fragment
	analysis rev	cagatggcggtcaacgtccagctaaccc	

For the quantification of genome copy numbers, cells of the isolates YI80-1, YI80-4 and YI80-5 were grown in 30 ml of MGM in 100 ml Erlenmeyer flasks at 37°C with a rotary frequency of 200 rpm. The cell densities were determined microscopically using a Neubauer counting chamber. For the preparation of cell extracts, aliquots of 3×10^8^ cells were withdrawn from cultures both in exponential and stationary phase, and harvested by centrifugation (5 min, 13000 rpm). The pellets were resuspended in 100 µl of 20% SW and the cells were lysed by addition of 900 µl sterile water. The lysis efficiency was determined microscopically and was nearly complete. The cell extracts were dialyzed against distilled water (MF-Millipore, 13 mm diameter, 0.025 µm pore size, VSWP01300). Serial dilutions were generated (1∶5, 1∶10, 1∶20, 1∶100) and 5 µl aliquots were used as real-time PCR templates.

As a prerequisite to generate standard curves for ploidy level quantification, fragments of ∼1 kbp of the *radA* gene were amplified using standard PCRs with cell extracts of the three isolates YI80-1, YI80-4, and YI80-5. The sequences of the oligonucleotides are listed in Table 1. The PCR fragments were purified by using preparative agarose gel electrophoresis and a gel extraction kit (Axygen Biosciences). DNA concentrations were determined photometrically using a photometer (ND-1000, Nanodrop Tech., Rockland, USA). The number of DNA molecules per volume was calculated using the molecular weights of the PCR fragments computed with “oligo calc” (www.basic.northwestern.edu/biotools) and the Avogadro number. For each standard fragment, a dilution series was generated and used for real-time PCR analysis in parallel with dilution series of the respective cell extract. The “analysis fragments” were about 200–300 bp and the oligonucleotides used are summarized in Table 1. The real-time PCR analyses were performed as previously described (29). The PCR conditions were 10 min 96°C, 50 cycles with 30 s 96°C, 30 s 65°C, 30 s 72°C followed by 5 min 72°C and a melt curve analysis from 62°C to 96°C in 1°C steps. By comparison of the threshold cycle (C_T_) differences of the different dilutions, it was verified that the PCR was exponential at least up to the threshold DNA concentration used for the analysis (i.e., a 10-fold dilution corresponds to a C_T_ difference of ∼3.32). In addition, a control with no template was included to ensure that product formation was based on the added template DNA in standard curve and the dilutions of cytoplasmic extracts. Furthermore, correct product formation and absence of byproduct was monitored by the generation of melting curves. A standard curve was generated and used to calculate the genome copy numbers present in the dilutions of the cell extracts. In each case, three biological replicates were performed. For every biological replicate, four dilutions of the cytoplasmic extracts were analyzed in duplicates, therefore, the calculated average ploidy levels rest on 24 technical replicates. The genome copy numbers and the cell densities were used to calculate the average genome copy numbers per cell and their standard deviations.

## Results

### Liquid pockets were observed in the crystal

**Figure 2:**
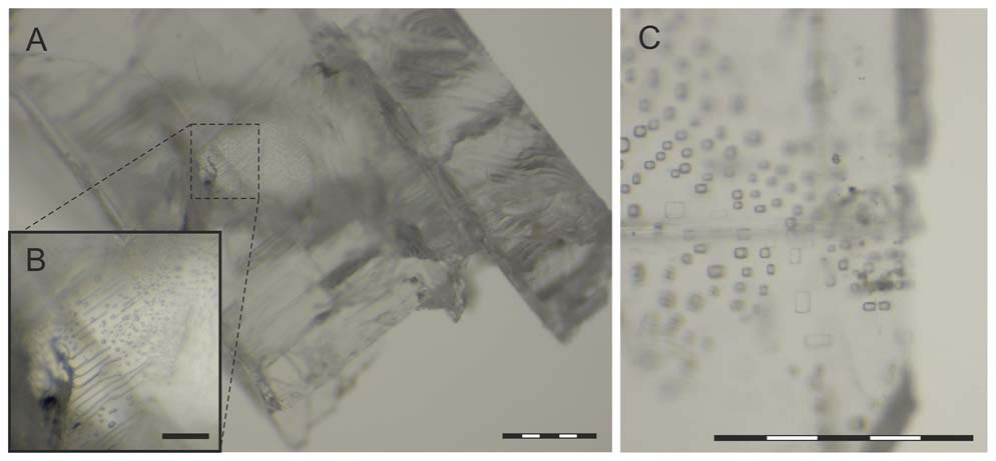
Crystal samples from 800 m visualized by light microscopy. (A) A salt crystal chiseled off from the surface sterilized isolation sample. (B) A magnification of a subsection of (A), showing liquid inclusions inside the crystal. (C) Cubic liquid inclusions. Bars represent 0.5 mm in A and C, and 0.1 mm in B.

The drill core (Fig. 1B), from which the halite samples were derived from, was drilled from Yunying salt mine Gaoyan Formation (Fig. 1A). Our samples were from the depths of 600 (Fig. 1C) and 800 m (Fig. 1D), where halite was deposited. The 600 m sample had a grainy structure, and had only few liquid pockets (data not shown). The 800 m crystal was clear and no cracks were visible. It had a euhedral form typical of primary crystals, and presence of cubic liquid inclusions was observed by light microscopy (Fig. 2). The inclusions were small and abundant, thus likely to have a syndepositional origin. The diameter of the largest inclusions was around 50 µm, but most of them were smaller, around 4–20 µm. No large (∼200 µm) inclusions indicative of postsedimentary recrystallization were detected.

### Species of *Halobacterium* and *Halolamina* were isolated

**Figure 3:**
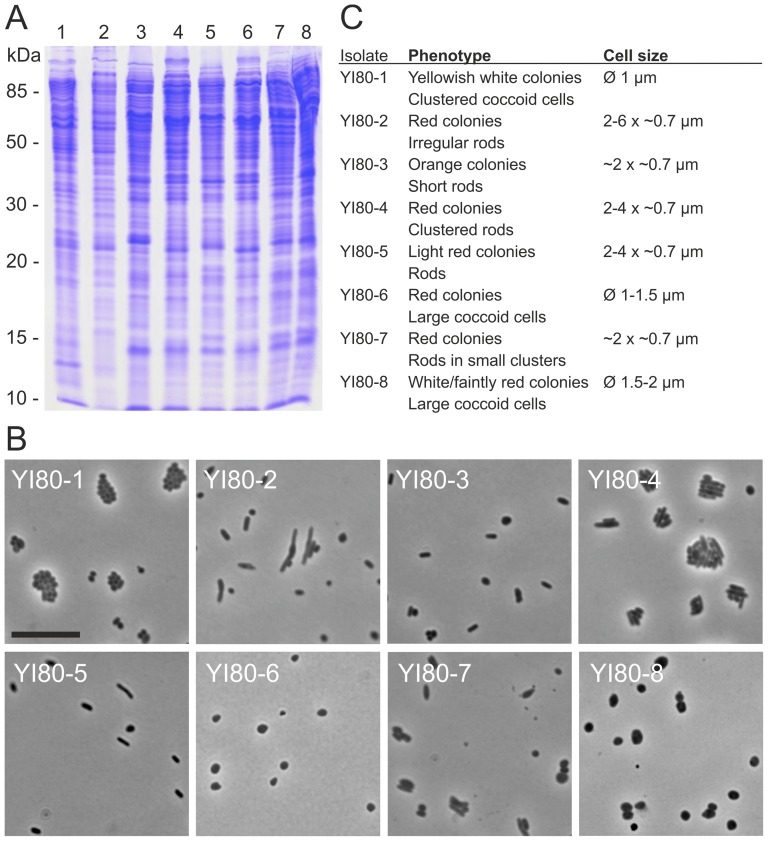
Microbes isolated from rock salt drill core sample from the depth of 800 m. (A) The whole cell protein analysis of the isolates by SDS-PAGE. The numbers refer to strains YI80-1 to YI80-8. Molecular mass markers are shown on left. (B) Light microscopy pictures of the isolates in late stationary phase. The scale bar in YI80-1 represents 10 µm, and is applicable to all pictures. (C) Phenotypic characteristics of the isolates.

Sterilization of equipment and solutions were shown to be efficient by sterility control platings resulting in no colonies. No growth originated from the 600 m crystal, but red and white colonies of different sizes were growing on plates with the 800 m sample. Repeated platings using other subsamples collected from the crystals gave similar results, proving our surface sterilization was effective. All plates were incubated for three months to detect all cultivable species from our samples. A few colonies of each phenotype derived from the 800 m sample were selected for further analysis. We obtained eight unique isolates with differing whole cell protein patterns (Fig. 3A). They were assigned as YI80 strains (YI80-1 to YI80-8; Table 2), YI referring to Yingcheng and 80 to 800 meters. Light microscopy revealed that the cells were either rod-shaped (YI80 strains 2–5 and 7) or coccoid (YI80 strains 1, 6, and 8) (Fig. 3B). Cells of YI80 strains 1, 4, and 7 grew in clusters (Fig. 3B). Characterization of the cells and colonies is given in Fig. 3C.

**Table 2:** Archaeal strains isolated in this study.

Strain	16S rRNA gene partial sequence GenBank Acc. No. and length (bp)
*Halobacterium* sp. YI80-1	KJ917623 (1386)
*Halobacterium* sp. YI80-2	KJ917624 (1396)
*Halolamina* sp. YI80-3	KJ917625 (1415)
*Halolamina* sp. YI80-4	KJ917626 (1376)
*Halolamina* sp. YI80-5	KJ917627 (1370)
*Halolamina* sp. YI80-6	KJ917628 (638)
*Halolamina* sp. YI80-7	KJ917629 (577)
*Halolamina* sp. YI80-8	KJ917630 (581)

**Figure 4:**
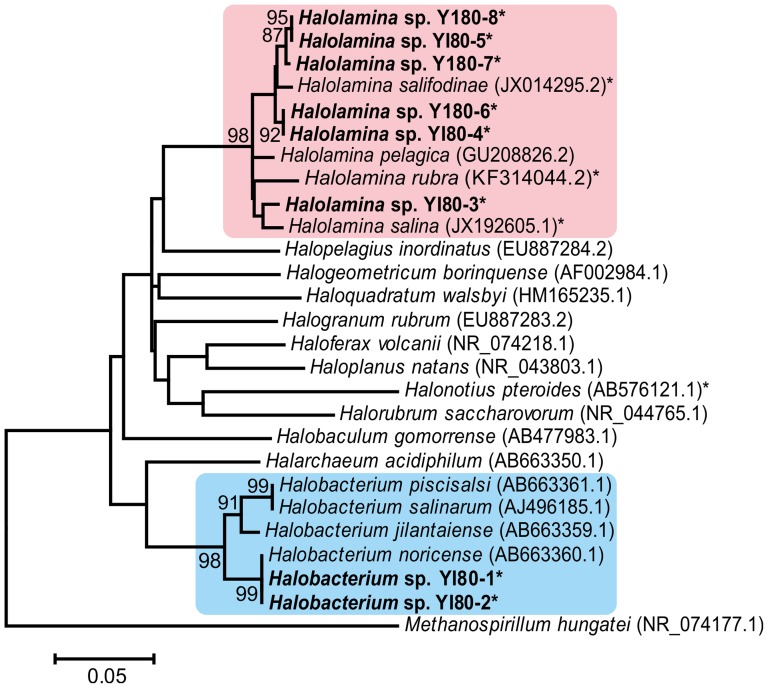
Maximum likelihood phylogenetic tree of the 16S rRNA gene sequences. GenBank numbers are given in brackets. *Halolamina* species are marked with a pink background, and species of *Halobacterium* with light blue one. Strains isolated from the 800 m rock salt sample are bolded, and partial sequences marked with an asterisk (*). Bar represents inferred substitutions per nucleotide position. *Methanospirillum hungatei* is used as an outgroup.

The 16S rRNA genes of the isolates were partially sequenced, and phylogenetic analysis was carried out (Fig. 4). The partial 16S rRNA gene sequences of YI80-6, YI80-7, and YI80-8 were significantly shorter (covering ∼600 bp of the 5′ end of the gene) than the other sequences (Table 2.), but aligned with the other sequences with ClustalW. Both maximum likelihood (Fig. 4) and maximum parsimony (data not shown) analyses showed that two of the isolates (YI80 strains 1 and 2) belong to the genus *Halobacterium*, and six of them (YI80 strains 3–8) to *Halolamina*. *Halobacterium* sp. YI80-1 and YI80-2 had identical 16S rRNA gene sequences, and were closely related to *Hbt. noricense* A1^T^, a Permo-Triassic isolate from the Alpine region [4]. The identity of the 16S rRNA gene sequences was 99.9%. All of our *Halolamina* isolates, except for YI80-3, grouped together and were most closely related to *Halolamina salifodinae* WSY15-H1^T^ with 97.4–98.8% 16S rRNA gene sequence identities (Fig. 4). Based on the 16S rRNA gene sequence, the closest match for YI80-3 was *Halolamina pelagica* TBN21^T^ with 97.9% identity. In the phylogenetic analysis, *Halolamina* sp. YI80-3 grouped with *Halolamina salina* (97.6% identity). The 16S rRNA gene sequences of the *Halolamina* isolates were 96.5–99.8% identical with each other.

### Ploidy levels of three *Halobacterium* and *Halolamina* isolates

**Figure 5:**
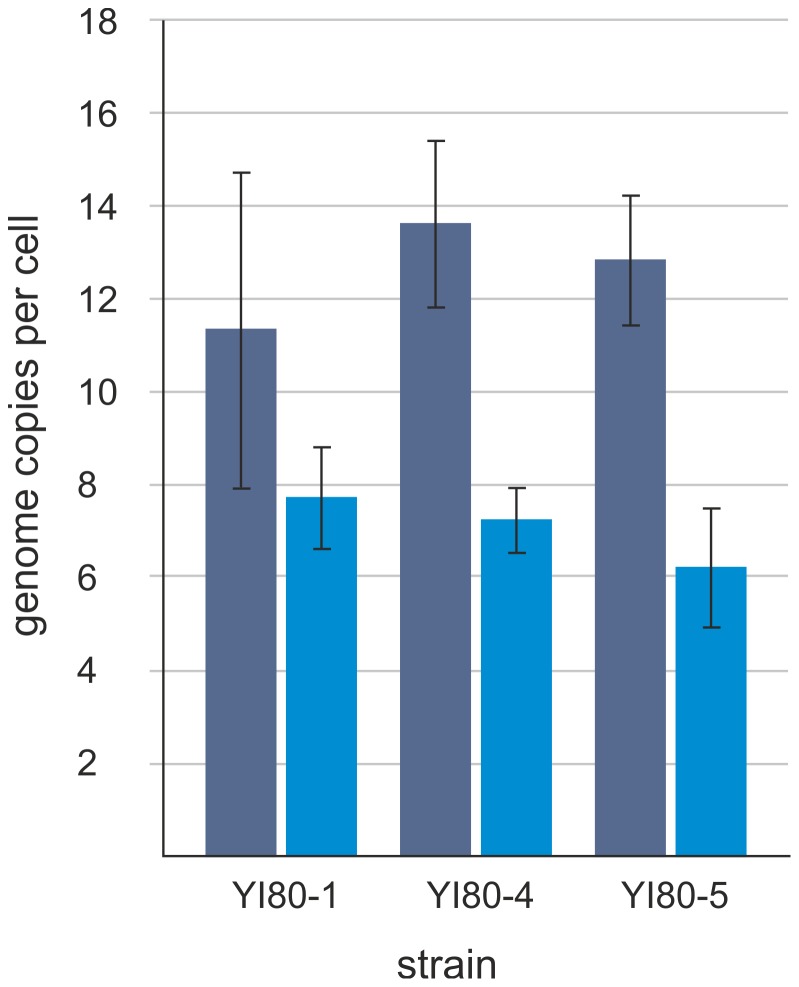
Ploidy levels of the isolates. Average copy numbers of chromosomes per cell during exponential (dark blue) and stationary (light blue) growth phases in *Halobacterium* sp. YI80-1, and *Halolamina* sp. YI80-4 and YI80-5, as detected by real-time PCR.

*Halobacterium* sp. YI80-2 grew very slowly; therefore, ploidy level quantification was restricted to *Halobacterium* sp. YI80-1, *Halolamina* sp. YI80-4, and *Halolamina* sp. YI80-5. In all three cases, the amplification of ∼1 kb-fragment of the single copy gene *radA* with degenerate primers was successful and yielded the sequence information necessary to apply the real-time PCR method for genome copy number determination. For each isolate, three independent cultures were grown and aliquots were removed at mid-exponential growth phase and during stationary phase. The average genome copy numbers and their standard deviations were determined and are shown in Fig. 5. All three isolates turned out to be polyploid during exponential growth and had average genome copy numbers of 11–14 per cell. In addition, in all cases, the average genome copy numbers were lower in stationary phase, and resting cells contained on average 6–8 genome copies per cell. While the number of three analyzed isolates is low, the high similarity of the results for three species of two different genera indicates that haloarchaea enclosed in ancient salt deposits might be typically polyploid, as has been hypothesized earlier.

## Discussion

It is not a generally appreciated fact that many microbes are polyploid. Multiple copies of a chromosome make cells more resistant towards DNA breakage and mutations, as all of the copies can be maintained identical [33], [38]. This could also mean an enhanced tolerance towards harsh living conditions. Halite burial sites, despite nutrient scarcity and lack of oxygen, are inhabited by bacteria and archaea that can be cultured in laboratory conditions [1]–[9]. The age and origin of these isolates have been debated [20], [57], [58]. If these microbes have indeed been buried inside halite deposits during sedimentation, the oldest viable cells known would be from Permian era, around 225–280 MYA [1], [4], [5]. To achieve an age of this magnitude, the cells need to be extremely well protected against DNA damage, or have to have the ability to repair DNA damage very effectively. The repair of the most severe DNA damage, i.e. the occurrence of many double strand breaks, resulting in high fragmentation of the chromosome, is discussed below. Notably, monoploid species are unable to regenerate intact chromosomes from shattered fragments. Overlapping genomic fragments are required, which are produced only by the fragmentation of chromosomes in polyploid species. The ability to repair shattered chromosomes would solve the contradiction between the claims that live haloarchaea have been isolated from ancient halite deposits and the major counterargument, i.e. that the stability of DNA is simply not high enough to survive such large time spans in an intact form.

Even with considering a postsedimentary origin for the cells, it would be unlikely for living microbes to be able to access deep underground halite deposits within a short time. Thus, with either syndepositional or postsedimentary origin, the age of the microbes would have to be high, exceeding the age of any other known living organisms. This led us to hypothesize that microbes retrieved from bedded rock salt would be polyploid. For this, we isolated cells of *Halobacterium* and *Halolamina* from a surface sterilized drill core sample from a site that has not been used for microbe isolation before. Our two halite samples from 600 and 800 m were from middle-late Eocene, around 40 million years old. The 800 m sample gave rise to colonies of several different phenotypes, in contrast to the 600 m sample from which nothing could be cultivated. This result is in accordance with observations of sporadic occurrence of viable microbes inside bedded halite [6], [8]. It also shows that our sterilization methods were sufficient, and the isolates do originate from inside our samples. Microbes have been found to be entrapped inside liquid inclusions [7], [17], which were abundant in the 800 m sample. The paucity of liquid inclusions in the 600 m crystal is likely related to no cultivable cells being obtained from this depth.

Among the seven genera, *Haloarcula*, *Halobacterium*, *Halococcus*, *Haloterrigena*, *Natronobacterium*, *Natronomonas*, and *Virgibacillus*, whose representatives have been isolated from ancient rock salt formations, species of *Halobacterium* are notably common [2], [4], [6], [7], [9], [18], [59]. They are known to be exceptionally resistant towards DNA damage and cellular stress [6], [60]–[62], which might be related to polyploidy [29], [34]. *Halobacterium* cells are typically rod shaped with red pigmentation [4], [63]–[65], but the isolated *Halobacterium* sp. YI80-1 grew in clusters of cocci, and the colonies were white. Species of the genus *Halolamina* have not been isolated from ancient halite deposits previously. Cells of *Halolamina* species are irregularly shaped [66], [67], except for *Halolamina rubra*, which is rod-shaped in liquid media and coccoid on solid surfaces [68]. Here, *Halolamina* isolates were either rod-shaped or coccoid, and coccoid cells were occasionally seen among rods. It is possible that some viable cells in the samples could not be cultured in the used conditions. Despite the anaerobic conditions present in the liquid inclusions, no attempts at anaerobic cultivation were made, since haloarchaea are known to be capable of both aerobic and anaerobic growth.

We selected two representatives of both genera of our isolates to be tested for polyploidy, but only three of them grew well enough to be analyzed. As all three analyzed isolates were polyploid, it suggests that polyploidy might be a common trait among halophilic archaea isolated from rock salt of great age. The *radA* gene was chosen to quantify the ploidy level of the three new isolates, because the gene is universally distributed in all three domains of life (*radA* in archaea, *recA* in bacteria and *rad51* in eukaryotes), and the degree of conservation is high enough to amplify a large fraction of the gene using degenerate primers. In addition, it is a single copy gene in prokaryotes with sequenced genomes, and thus it is ideally suited for the determination of the ploidy level. It should be noted that this approach of using one gene with an unknown localization on the chromosome would not be feasible for fast growing mero-oligoploid species. For example, *E. coli* cells growing under optimal conditions with a doubling time of 25 minutes contain an average number of 6.8 origins and 1.7 termini [30], and thus the copy number of a gene is highly influenced by its chromosomal localization. In *E. coli* culture growing with a doubling time of 103 minutes, the difference is far less pronounced, they contain on average 2.5 origins and 1.2 termini. However, haloarchaea, including the three new isolates, grow much slower and have doubling times of 3–4 hours under optimal conditions. Therefore, it can be expected that the impact of the chromosomal localization on the copy number of a gene is much lower in haloarchaea than in slow growing *E. coli* cultures. Indeed, for *Hbt. salinarum* it has been verified that the copy numbers of five sites, which were evenly distributed around the chromosome, were identical throughout different growth phases [69]. Another possible caveat of quantifying the copy number of one gene using the real-time PCR approach might be that the method might lead – for unknown reasons – to erroneous results. However, the method has been compared with various different, independent approaches in previous studies, e.g. quantitative Southern blotting, spectroscopic quantification of the DNA content, and the wealth of approaches that have been used to characterize the chromosome of *E. coli* [29], [30], [33]. In all cases, the results of the real-time PCR approach were in agreement with that of independent approaches showing that the method is sensitive and has a high precision.

Polyploidy could also be typical for haloarchaea in general, as the three previously characterized species isolated from current environments have been found to be polyploid, i.e. *Hbt. salinarum*, *Hfx. volcanii*, and *Hfx. mediterranei* [34]. However, these three species from two genera have been cultivated in laboratory conditions for decades; *Hfx. mediterranei* and *Hfx. volcanii* were isolated 30 and 40 years ago, and *Hbt. salinarum* was isolated about 100 years ago. Therefore, to our knowledge, we report here for the first time the ploidy levels of haloarchaeal species that were freshly isolated. In addition, the third genus, *Halolamina*, is added to the haloarchaeal genera containing polyploid species, and the number of isolated *Halolamina* species is doubled.

Long-term survival over geological times is probably not the advantage that has led to the selection of this trait in haloarchaea. However, haloarchaea live in environments characterized by intense sunshine with high UV dose, high rates of water evaporation and salt accumulation. In these environments, periodical droughts are typical, necessitating an extremely high resistance against total desiccation in combination with high solar radiation including the UV part of the spectrum. Desiccation leads to DNA double strand breaks (DSBs), and thus species with a high capacity to repair DSBs have a high desiccation resistance and a high selective advantage in such environments. The mechanism of DSB repair has been best studied for the desiccation and irradiation resistant bacterium *Deinococcus* radiodurans, which can regenerate intact chromosomes from hundreds or thousands of scattered fragments [70]. It is a two-stage process that involves DNA synthesis followed by recombination. The short-term resistance of total desiccation has been verified for *Hbt. salinarum* as well as for *Hfx. volcanii* [35], [62]. In addition, it has been shown that haloarchaea can survive in desert rocks, a very extreme terrestrial environment [71]. Desiccation resistance is not the only evolutionary advantage of polyploidy. Various evolutionary advantages with experimental evidence for at least one haloarchaeal species have recently been summarized [69]. Notably, recently it was shown that genomic DNA can serve as an intracellular phosphate storage polymer for *Hfx. volcanii*, which is able to grow in the absence of any external phosphate source [35]. Thus *Hfx. volcanii* and probably also other polyploid haloarchaea can grow in phosphate-free environments, in which growth for species without a phosphate storage is impossible.

The high selective force to survive extreme terrestrial conditions of various kinds that are present today and were identically present already billions years ago can explain why polyploidy evolved in haloarchaea. One by-product of this selection is the ability to survive over geological times if entrapped in a halite deposit. Even more surprising by-products are the abilities to survive real extra-terrestrial conditions of space flights or simulated Martian conditions [72], [73]. Put together, the every-day terrestrial conditions that haloarchaea have to face are so extreme that they have enforced the evolution of survival strategies, including polyploidy, that enables them to survive even rather bizarre challenges like exposure to extraterrestrial conditions or survival over geological times entrapped in halite deposits.
